# Theta-Defensin Proteins: Conformational Variability and Environmental Effects

**DOI:** 10.34133/csbj.0182

**Published:** 2026-07-28

**Authors:** Nicolas Vatiliotis, Finn van Loon, Doğa Selin Damar, Maria Fyta

**Affiliations:** ^1^ Computational Biotechnology, RWTH Aachen University, 52074 Aachen, Germany.; ^2^ Molecular Biology and Genetics, Istanbul Technical University, 34469 Istanbul, Turkey.; ^3^ Center for Computational Life Sciences (CCLS), RWTH Aachen University, 52072 Aachen, Germany.

## Abstract

Theta-defensins are a unique class of cyclic antimicrobial peptides known for their exceptional structural stability and broad-spectrum antimicrobial activity. Despite their therapeutic potential in mammals, the effects of solvent environments and specific mutations on their conformational stability remain poorly understood. This paper employs all-atom molecular dynamics simulations under different solvent conditions to investigate the structural dynamics of baboon theta-defensin-2 (BTD-2) peptides, as well as solvent effects. BTD-2 was selected due to its higher arginine content, enhancing its antimicrobial potency compared to that of the rhesus theta-defensin-1 protein. We compare the dynamics of 3 different BTD-2 conformations and assess their flexibility, residual fluctuations, and thermal stability. Although similar in sequence, these peptides provide conformation-specific temporal characteristics. The insights gained through the analysis of the simulation data contribute to a deeper understanding of theta-defensin stability, providing important information related to the rational design of peptide-based antimicrobial and antiviral therapeutics.

## Introduction

Theta-defensins [1–4] are a unique class of cyclic antimicrobial peptides characterized by their distinct structural properties and broad-spectrum antimicrobial activities [5]. Initially discovered in the leukocytes of rhesus macaques [6], theta-defensins remain the only known cyclic peptides in mammals and could harbor great potential [7]. Their special feature is a cyclic cysteine ladder motif, formed by 3 parallel disulfide bonds, which imposes remarkable structural stability and resistance to proteolytic degradation [8]. The discovery of theta-defensins represented an important milestone in understanding the innate immunity of Old World monkeys and certain ape species, as these cyclic peptides exhibit unique structural stability and antimicrobial properties, providing insights into their host defense mechanisms [9]. The first identified theta-defensin, rhesus theta-defensin-1 (RTD-1), was reported in 1999, and subsequent research has highlighted its potent antimicrobial activity against a wide range of pathogens, including bacteria, fungi, and viruses [6]. Other theta-defensin variants, such as baboon theta-defensin-2 (BTD-2), have also been characterized, sharing the conserved cyclic cysteine ladder structure essential for their stability and function [8].

Their cyclic nature not only enhances their resistance to enzymatic degradation but also facilitates their self-association into structured assemblies, a feature that may be leveraged for the design of stable peptide-based therapeutics [10,11]. In fact, the lack of defensins in mice’s neutrophils has raised doubts about the use of mice as a faithful model of human innate immunity [12]. Nevertheless, despite their potential, critical aspects of theta-defensin function remain unresolved, particularly their behavior under varying environmental conditions and the impact of structural modifications. Specifically, the influence of different molecular force fields and solvent environments on the conformational stability and dynamic properties of theta-defensins has not been extensively studied. Addressing these knowledge gaps is essential to advancing the development of defensin-based antimicrobial and antiviral agents [8]. Recent studies provide a useful broader context for this point. In antimicrobial peptide research, prediction-based approaches can be limited when conformational flexibility and membrane-associated structural changes are not considered explicitly [13]. Along similar lines, studies combining molecular simulations with experimental structural data have shown that static structures do not always capture the conformational behavior observed in solution [14]. Mutation-focused molecular dynamics (MD) studies further show that a peptide or protein can retain its overall fold while still displaying altered dynamical behavior [15]. These findings support the use of atomistic simulations to study theta-defensins not only as highly stable cyclic scaffolds but also as dynamic molecular systems whose behavior may depend on sequence variation and environmental conditions.

Theta-defensins variations differ in structure, cysteine connectivity, and functional properties and are accordingly classified into 3 major subfamilies: alpha-defensins, beta-defensins, and theta-defensins, as characterized in Table 1. The first theta-defensin, RTD-1, was isolated from the leukocytes of rhesus macaques and identified [6]. Theta-defensins are exclusively found in Old World monkeys, such as rhesus macaques and baboons (*Papio anubis*), but are absent in New World monkeys and humans due to evolutionary divergence [16]. The name “theta-defensin” derives from the Greek letter *θ*, chosen due to the cyclic nature of these peptides, reminiscent of the circular shape of *θ*. Unlike linear antimicrobial peptides, their cyclic conformation provides exceptional structural integrity and resistance to proteolytic degradation. This unique feature contributes to their role in innate immunity—the body’s first line of defense against microbial pathogens.

**Table 1.** The classification of the 3 theta-defensin peptide types with respect to the number of disulfide bridges, amino acid residues, and their structural properties

Defensins	Disulfide bridges	Structure	Amino acid residues
Alpha	1–6, 2–4, 3–5	Triple-stranded β-sheet with 2 β-turns	29–35
Beta	1–5, 2–4, 3–6	Predominantly β-sheet	Up to 45
Theta	3 pairs in a ladder pattern	Cyclic octadecapeptide	18

Interestingly, while theta-defensin genes remain present in the human genome, they are non-functional due to a premature stop codon mutation at position 17 [16], preventing the proper translation of functional theta-defensins [16]. The absence of theta-defensins in humans has been hypothesized to increase susceptibility to infections, particularly retroviruses such as HIV-1. Despite this genetic loss, theta-defensins such as RTD-1 remain active in nonhuman primates, where they contribute to broad-spectrum antimicrobial defense by disrupting microbial membranes and inhibiting viral entry [8]. Along the purposes of the latter, mutations in theta-defensin peptides have been investigated for the treatment against HIV [17,18] or other viruses [19].

### Structural characteristics of BTD-2

**Fig. 1.**
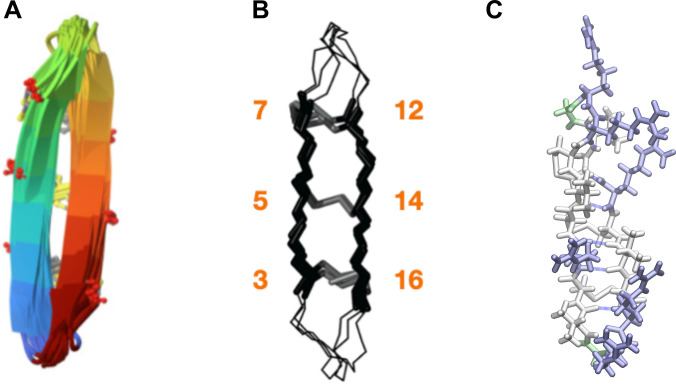
Three different representations of the baboon theta-defensin-2 (BTD-2) peptide: (A) a sketch of the cyclic conformation coarsely coloring each residue position, (B) a wireframe representation with the numbers corresponding to the cysteine (CYS) residues involved in the 3 disulfide bonds (also shown) providing the ladderlike connectivity pattern, and (C) an all-atom representation colored with respect to the amino acid type (green: GLY; blue: ARG; white: CYS).

BTD-2 is a structurally distinct antimicrobial peptide catalogued under Protein Data Bank (PDB) entry “2LYE”. This peptide features a cyclic peptide backbone, formed via head-to-tail cyclization of its amino acid sequence. Such a conformation is critical for maintaining BTD-2’s structural integrity, reducing enzymatic degradation, and ensuring its functional stability in biological environments. BTD-2 consists of 18 amino acids, arranged in a circular configuration and stabilized by 3 disulfide bonds. These bonds follow a ladderlike connectivity pattern—Cys3–Cys16, Cys5–Cys14, and Cys7–Cys12, as illustrated in Fig. 1. This parallel arrangement importantly enhances the peptide’s rigidity and stability, ensuring that it retains its bioactivity in diverse physiological conditions [9]. BTD-2 is a homodimer, consisting of 2 identical peptide units that associate symmetrically. This symmetry has been confirmed through nuclear magnetic resonance (NMR) spectroscopy, revealing a highly ordered and stable structure [20]. Dimerization enhances BTD-2’s ability to interact with microbial membranes, optimizing its antimicrobial function [21]. The stability of BTD-2 is largely attributed to its cyclic backbone and cysteine ladder motif, which render it highly resistant to enzymatic degradation. This resilience enables BTD-2 to function effectively under extreme conditions where linear antimicrobial peptides may lose activity. Moreover, its unique structure contributes to its ability to inhibit viral infections by interfering with pathogen entry mechanisms [9,21]. Despite important advances in theta-defensin research, several fundamental questions remain unanswered. While the cyclic structure and antimicrobial properties of theta-defensins have been well-documented, their behavior at the molecular level under different environmental conditions is not fully understood [8,22].

In order to fill this knowledge gap hindering the ability to optimize theta-defensins for therapeutic applications, as well as the development of peptide-based antimicrobial and antiviral agents [22], we perform atomistic simulations tackling 2 primary research questions: (a) How do different solvent environments influence the structural stability and dynamic behavior of BTD-2? (b) What is the effect of specific mutations on the conformational stability of this peptide? This work aims to systematically investigate the structural responses and dynamic behavior of BTD-2 using atomistic simulations. Specifically, we assess the impact of 3 different electrolyte solutions and thermal fluctuations on the stability and structural dynamics of BTD-2, providing essential insights into environmental and evolutionary effects on the cyclic nature of the theta-defensin conformation in view of fundamental peptide science and potential biotechnological applications.

## Methodology

### MD simulations

For the simulations of the structural dynamics of BTD-2, MD simulations within the GROMACS [23] implementation are performed. The CHARMM36 force field [24] was used for the protein and the electrolyte together with the TIP3P explicit water model [25]. The BTD-2 peptide was selected due to its increased number of arginine residues compared to that of RTD-1, enhancing its antimicrobial potency. Additionally, BTD-2 exhibits a homodimeric structure with axial symmetry, providing an excellent model for studying cyclic peptides in innate immunity. In order to assess the structural response of BTD-2 focusing on stability, flexibility, and intermolecular interactions to different solvent conditions, we model the protein in different electrolyte solutions and temperatures. For the solvent, 3 different electrolytes were modeled, namely, the standard physiological sodium chloride (NaCl), potassium chloride (KCl), and the denaturant guanidinium chloride (GdmCl). For NaCl and KCl, the concentration was varied from 0 to 1.5 M. GdmCl was considered at a 1 M concentration. These 3 solutions are representative of simple monovalent salt and denaturant solutions with the aim of providing a first indication of the role of different solvents in peptide stability and response to the environment to initiate follow-up studies choosing the solvent in a more directed, designed way toward specific applications.

**Fig. 2.**
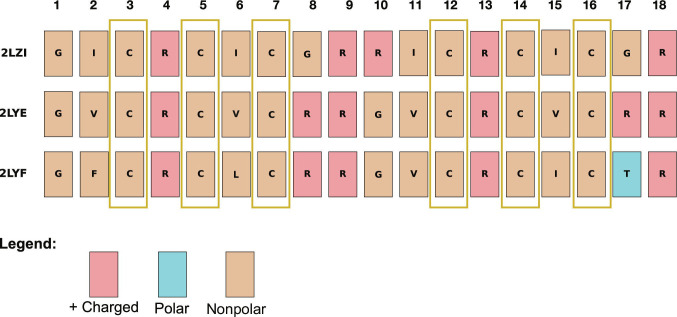
Multiple sequence alignment of the 3 related peptides human theta-defensin-2 (HTD-2), baboon theta-defensin-2 (BTD-2), and rhesus theta-defensin-1 (RTD-1) (Protein Data Bank [PDB] IDs respectively: 2LZI, 2LYE, and 2LYF). The labels describe the amino acid type using the standard one-letter nomenclature. The colors correspond to the charge state as provided in the legend. The index of each amino acid is also shown on the top.

The initial structures of the 3 available theta-defensin structures, namely, BTD-2 (PDB: 2LYE [26]), RTD-1 (PDB: 2LYF [27]), and the human theta-defensin-2 (HTD-2; PDB: 2LZI [28]), were obtained from the PDB and modeled. For all simulations, we used the same setup. Although the structures of the 3 peptides are very similar, small differences at the residue level are observed, as sketched in Fig. 2. The index of the amino acids is also provided and can be used as a reference for the discussion in the following. The alignment sketched highlights regions of sequence conservation. The complete conservation of all cysteine (C) residues is evident, along with highly conserved glycine (G) and arginine (R) residues (indicated by dark red shading), suggesting a common structural motif and shared functional properties [29].

The peptides were separately placed in a cubic simulation box with a minimum distance of 1.5 nm from the edges to avoid self-interactions due to periodic boundary conditions. In order to efficiently model the protein and optimize all interactions and distances, an energy minimization was performed using the steepest descent algorithm followed by 2 equilibration steps of 10-ns duration each, one under the (NVT) ensemble using a velocity-rescaling thermostat [30] and one under the (NPT) ensemble using a Parrinello–Rahman barostat [31]. The subsequent production runs were performed for 500 ns within the same (NPT) ensemble. In this step, the simulation was performed with a time integration of 2 fs, with a compressed output of 5,000 trajectories, used for later analysis. The final 100 ns of the trajectories were used for the calculation of the averages and assessment of structural stability and conformational dynamics, residue flexibility, and protein–solvent interactions. Note that for the analysis of the MD trajectories, we follow the common path of other protein simulations [32–35]. In order to more quantitatively assess the statistics in the MD observables, methods such as block averaging could support the MD findings on sequence/structure-based effects [36,37]. The barostat was set to a target pressure of 1 bar with a coupling parameter *τ_p_* of 5 ps; the thermostat was set to 303.15 K with a coupling *τ_T_* of 1 ps. The simulation box size during the production simulations varied between 5.1 and 5.6 nm due to the isotropic volume rescaling. Across all simulations, of 1 M solvent concentration, around 18,500 atoms were simulated, where 282 of these were protein atoms.

### Analysis of simulation data

In order to analyze the structural stability, dynamics, solvent effects, and local environment of the 3 different peptides, we calculated the most relevant properties. For the influence of the solvent environment, the solvent-accessible surface area (SASA) was calculated to determine peptide–solvent interactions and structural rearrangements. The mean square displacement (MSD) provided insights into peptide mobility across different solutions, while the radial distribution functions characterized peptide–solvent and ion interactions, revealing hydration patterns and binding effects. The root mean square deviation (RMSD) was used to quantify global structural stability, while the root mean square fluctuation (RMSF) provided residue-level flexibility insights. Changes in hydrogen-bonding patterns were examined to assess their role in maintaining peptide conformation.

On top of these properties and in order to uncover hidden information, we also perform an unsupervised machine learning analysis of the simulation data. For this, we resort to dimensionality reduction techniques. A common linear scheme that can capture essential motions and conformational changes of proteins as calculated through MD simulations is principal component analysis (PCA). The essence of PCA is that it will return a *k*-dimensional subspace while maximizing its variance. This can be achieved by calculating *k* eigenvalues with the largest eigenvalues of the covariance matrix *C* of an initial set of feature vectors. In this case, the inputs are the trajectories of the backbone C_α_ atoms in cartesian space. In an effort to compare the different solvents, the trajectory data are preprocessed to eliminate overall translation and rotation of the protein to focus on conformational changes. The origin of the system is set to the center of mass of the protein. The data are then projected on to the principal component (PC) vectors by *Z* = *X* · *A*, where *A* is the matrix of the eigenvectors. Before employing PCA, the trajectory data are arranged in a matrix of a dimension equal to the number of frames multiplied to 3 · *N*, where *N* is the number of atoms [38]. PCA is particularly useful for visualizing how solvent environments influence peptide movement, revealing critical conformational changes [39].

In order to also capture non-linear correlations, the non-linear dimensionality reduction algorithm uniform manifold approximation and projection (UMAP) is also applied on the simulated trajectories. UMAP uses local manifold approximations to calculate a topological representation of the high-dimensional data and the low-dimensional representation. This representation is then changed in order to minimize the cross-entropy between the 2 representations [40]. Fifteen neighbors were taken into account for the UMAP analysis with a minimal distance of data points in the low-dimensional representation of *min_dist* = 0.1 and a euclidean distance metric.

## Results and Discussion

We begin the discussion with one of the protein structures (ID 2LYE in the PDB) and the influence of the different solvents on this. In the next step, we compare the 3 available BTD-2 theta-defensin structures and evaluate temperature effects.

### Thermal stability under ionic stress

We begin the analysis, first focusing on one of the BTD-2 peptides with ID 2LYE to probe its stability under a broader range of environmental conditions, specifically varying the NaCl concentration and the temperature. To assess ionic tolerance, BTD-2 was simulated in solutions of 0, 0.1, 0.5, 1, and 1.5 M NaCl. The structural analysis revealed no important conformational changes across the [0:1] M range, indicating a high degree of structural integrity under moderate ionic variations. However, beyond 1 M NaCl, minor fluctuations were observed, suggesting the onset of ionic stress effects at higher concentrations. Given the stability of BTD-2 at 0.5 M NaCl, the effect of temperature (280, 290, 300, and 310 K) was tested at this concentration. The results indicated that at 0.5 M NaCl, temperature variations had a negligible impact on the peptide’s structure, confirming its robustness in this ionic environment. In order to stretch the dynamic stability of the peptide, the thermal stability analysis was extended to consider a 1 M NaCl solution. At all temperatures, fluctuations of almost the same range were observed in both the RMSD and the SASA at different temporal locations.

**Fig. 3.**
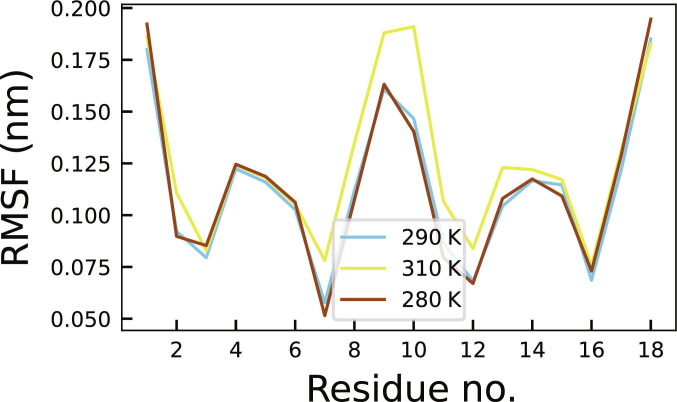
Root mean square fluctuation (RMSF) of baboon theta-defensin-2 (BTD-2) at 1 M NaCl. The peptide shows increased loop flexibility at 310 K. For the index, refer to Fig. 2.

**Fig. 4.**
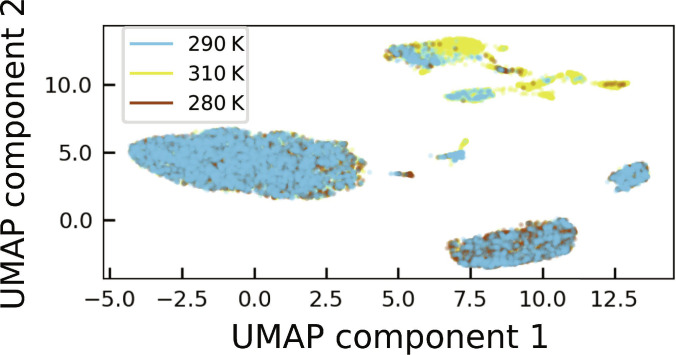
The uniform manifold approximation and projection (UMAP) representation of the protein trajectory during the 500-ns simulation of wild-type (WT) baboon theta-defensin-2 (BTD-2; ID: 2LYE) at different temperatures, as denoted in the legend.

The residue-specific flexibility was analyzed using RMSF in Fig. 3). As expected, the highest temperature (310 K) correlates with the highest atomic fluctuations, particularly in the loop region (residues 8 to 10), indicating thermal destabilization of these flexible domains. In contrast, 280 and 290 K show reduced fluctuations in these regions, preserving a more rigid backbone structure. The UMAP representation of the protein trajectory throughout the simulations for the 3 temperatures are sketched in Fig. 4. It can be clearly seen that the conformational space of all temperatures is overlapping. A clear difference can be seen for the high temperature, which provides additional states (follow only yellow regions in the plot).

### Influence of the solvent

**Fig. 5.**
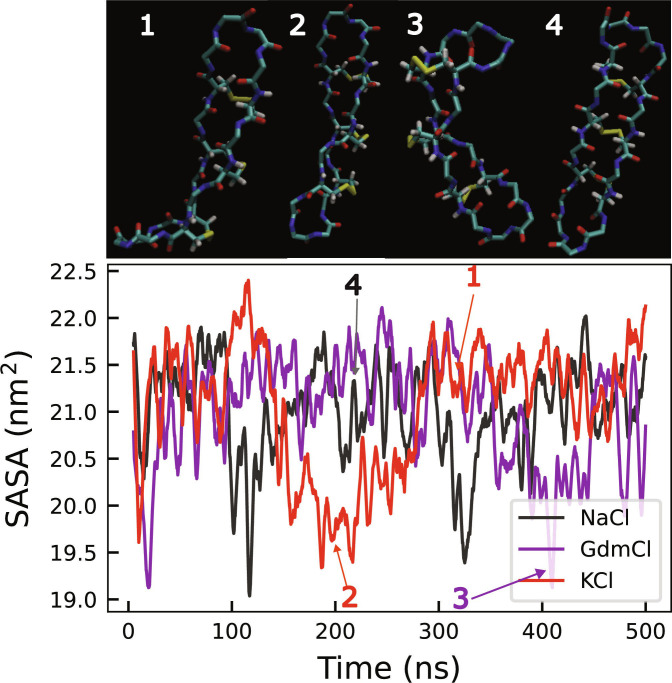
Bottom: moving average of the solvent-accessible surface area (SASA) of the baboon theta-defensin-2 (BTD-2; ID: 2LYE) protein over a 500-ns simulation in different solvent environments as described in the legend. Four selected conformations are shown on the top from the different simulations at different SASA values (see text).

We move on to analyzing the influence of different types of solvents through the SASA. This property qualitatively assesses the surface of BTD-2 exposed to the solvent molecules throughout the simulation and is provided in Fig. 5 for the 3 solvents. This quantity is quite sensitive to how tightly the peptide is packed, so even small shifts in its shape show up quickly in the SASA curve. In the 3 solvent systems (NaCl, KCl, and GdmCl) shown in Fig. 5, the values fluctuate but remain within roughly the same interval throughout the 500-ns run. The traces show the usual short drops and rises that appear when the backbone adjusts, but they return to the same level afterward. GdmCl is expected to broaden the exposed surface if the peptide begins to unfold. This effect is not visible here due to the cyclic nature of the peptide. GdmCl behaves almost like NaCl and KCl, and none of the solvents produces a persistent shift toward a higher SASA. Instead, the peptide keeps roughly the same accessible area across all conditions. This behavior is consistent with the known mechanical rigidity of BTD-2. The data here suggest that, at least within the simulated time scale, BTD-2 remains intact and is not noticeably destabilized by GdmCl. The 4 selected conformations in Fig. 5 provide qualitative structural comparison to NMR studies [8]. Conformation 1 in KCl reveals a tilted turn region at the bottom corresponding to a SASA of 21.13 nm^2^. Conformation 2 features a twisted conformation and a SASA of 19.46 nm^2^. Conformation 3 in GdmCl at a deep decrease with a SASA value of 18.47 nm^2^ corresponds at this timestep in a warping of the whole backbone structure. Conformation 4 in NaCl reveals a straight backbone and a SASA of 22.17 nm^2^. Overall, the snapshots qualitatively support the NMR insights on a stable and highly ordered protein structure [20], which is further confirmed by the following analysis on the RMSF and PCA revealing that the protein is robust against the solvent.

**Fig. 6.**
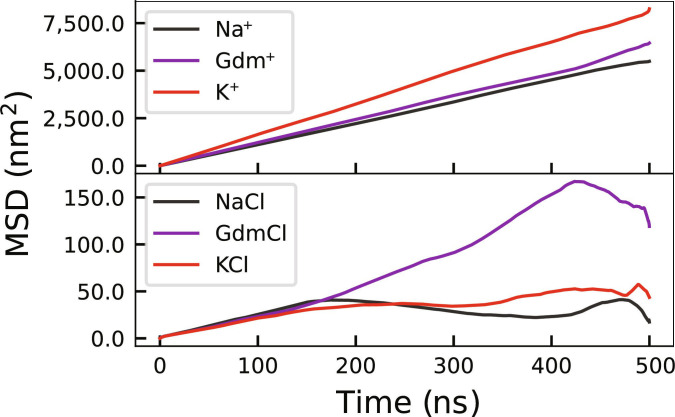
The mean square displacement (MSD) for (top) the ions and (bottom) the protein (ID: 2LYE) in the different solutions as described in the legend along the simulation time.

We next assess the mobility of all species in the solution through the MSD. For the cations (top panel) in Fig. 6, the MSD for all 3 solutions shows the expected ballistic and diffusion-like increase. The slope differs between the systems: K^+^ shows the largest increase, followed by Gdm^+^ and Na^+^. This difference reflects the typical mobility variations between these ions, with potassium generally diffusing faster in aqueous environments than sodium and guanidinium being the slowest due to its mass. The lower panel shows the MSD of the BTD-2 peptide (ID: 2LYE). In contrast to the ions, the protein moves on a much smaller scale and the traces separate more clearly between the solvents. In NaCl, the curve remains low and flat, indicating minimal drift and a relatively tight confinement of the peptide. In GdmCl, the MSD grows more strongly and reaches its maximum around 350 to 400 ns before leveling off. The KCl system lies between the other 2: it increases gradually but remains below the GdmCl values throughout the run. The shape of the protein MSD in GdmCl suggests that the peptide experiences slightly larger center-of-mass motion in this environment, although the absolute values remain small for a molecule of this size. Together with the structural analyses from the previous graphs, this behavior still falls within the range expected for a compact, cyclic peptide and does not point to any solvent-induced unfolding. Instead, the MSD mainly captures differences in how the entire peptide diffuses within each simulation box. The fluctuations revealed in the RMSD remain on a rather small scale for the simulation duration, pointing to small conformational changes around the initial conformation.

**Fig. 7.**
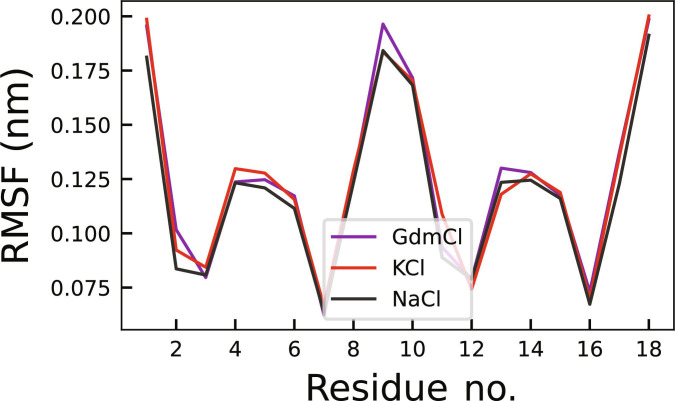
Root mean square fluctuations (RMSFs) of baboon theta-defensin-2 (BTD-2; ID: 2LYE) in the 3 solvents (see legend) over the 500-ns trajectory. For the index, refer to Fig. 2.

In order to unveil any local distortion, RMSFs are provided for the 3 solvents in Fig. 7. A first inspection of the curves reveals that all curves are very similar. The largest fluctuations occur at positions adjacent to the cyclization point and at the residues forming the β-turns, while the central parts of the β-strands remain comparatively stable. This pattern is characteristic of the cyclic cysteine ladder fold and reflects the intrinsic flexibility of these structural regions rather than any solvent-driven effect. No residue exhibits a consistent solvent-specific deviation. There is a small rise near residue 9 (an Arg) in the GdmCl trace. This is a very local fluctuation not affecting the neighboring residues. The NaCl and KCl lines run almost on top of each other along most residues, with only tiny shifts that are well within normal variation. From the shape of the profiles, the flexibility at each position seems to arise from the residue position in the peptide. None of the solvents appears to alter this pattern in a meaningful way, and the peaks line up with regions already known to be the more mobile parts of this peptide class.

**Fig. 8.**
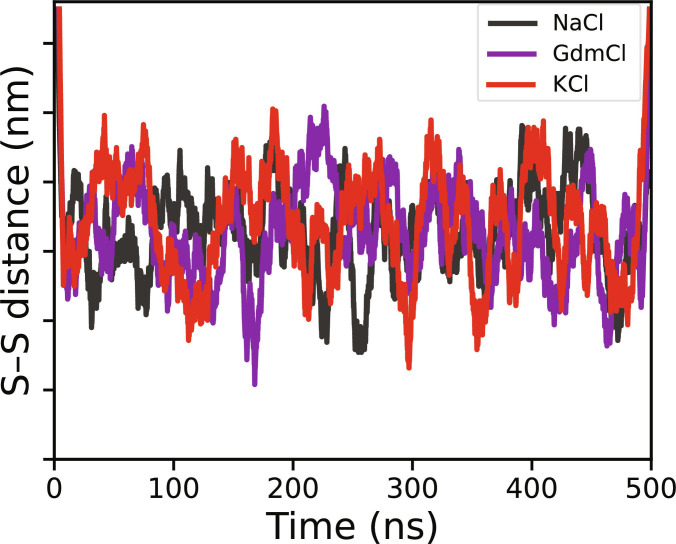
Stability of the central Cys5–Cys14 disulfide bridge of baboon theta-defensin-2 (BTD-2) in different solvent environments, as denoted in the legend, over 500-ns simulations.

In order to evaluate whether the solvent environment affects the covalent stabilization of BTD-2, the central disulfide bridge (sulfur–sulfur distance) was monitored over the full 500-ns trajectory (Fig. 8). In all 3 solutions, this distance fluctuates within the same range and shows similar dynamics. This behavior indicates that the central disulfide bridge is preserved throughout the simulations and stabilizes the protein. Interestingly, the denaturant solvent GdmCl does not impose a systematic drift or rupture-like behavior of the protein compared to NaCl and KCl. Accordingly, the solvent-dependent effects observed for BTD-2 should not be interpreted as disruption of the central disulfide bridge. Instead, they are more consistent with local rearrangements around a stable cyclic cysteine scaffold, especially at solvent-exposed and charged residues.

**Fig. 9.**
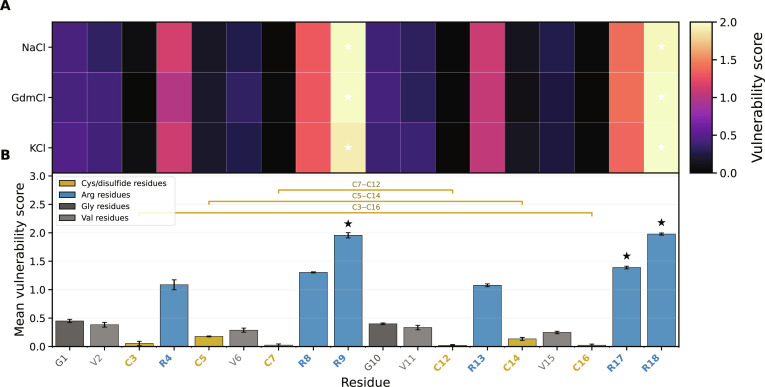
Residue-level vulnerability profile of baboon theta-defensin-2 (BTD-2) as described by (A) the residue-wise vulnerability map for BTD-2 in NaCl, guanidinium chloride (GdmCl), and KCl. In (B), the mean residue vulnerability score is averaged across the 3 solvent environments. The residues are color coded according to the residue type. Stars (*) indicate the residues with the highest vulnerability scores, identified as the principal vulnerability hotspots consistently observed across all solvent conditions.

We next assess the parts of BTD-2 that are most sensitive to the solvent environment. For this, a residue-level vulnerability score was calculated by combining the normalized residue flexibility and solvent exposure. The vulnerability score combines normalized residue flexibility and solvent exposure, allowing residues that are both mobile and solvent accessible to be identified. The vulnerability map in Fig. 9A shows a similar residue-wise pattern across NaCl, GdmCl, and KCl, suggesting that the solvent response is mainly controlled by specific positions rather than by global unfolding. The vulnerability score is depicted in Fig. 9B with respect to the residue position and type. Cysteine residues indicate the disulfide-stabilized scaffold; arginine and glycine/valine residues represent the dominant cationic sites and neutral backbone-supporting positions, respectively. The highest vulnerability score is observed at the arginine-rich regions, especially R9, R17, and R18, with additional contributions from R4, R8, and R13. These residues are solvent exposed and positively charged, making them likely interaction points with the surrounding ionic environment. In contrast, the cysteine residues involved in the disulfide ladder show low vulnerability scores. This indicates that the covalent scaffold remains comparatively protected, even when the surrounding solvent changes. Together with the disulfide-distance analysis, this supports a model in which BTD-2 preserves its cyclic cysteine core while solvent-dependent rearrangements occur mainly around exposed charged residues.

**Fig. 10.**
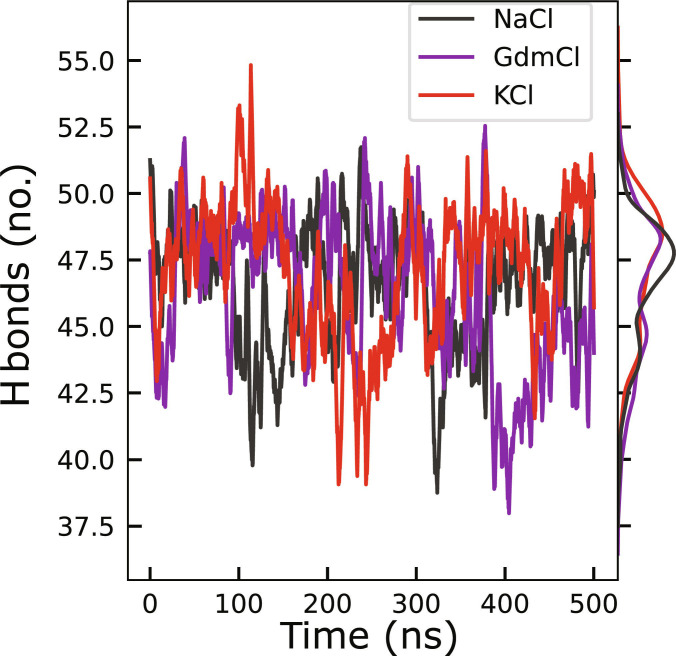
Time-series of the number of hydrogen bonds between protein and water. The histogram on the right depicts the respective distributions.

Together with the disulfide-distance analysis, the residue vulnerability profile suggests that BTD-2 preserves its cyclic cysteine core, while solvent-dependent rearrangements occur mainly around exposed charged residues. In order to assess whether this local solvent response is also reflected in the hydrogen-bonding pattern, the internal peptide hydrogen bonds and peptide–solvent hydrogen bonds were calculated and are summarized in Table 2. The reported values correspond to the standard error of the mean (SEM) from block averaging. Specifically, the MD trajectories were split into blocks of 50 ns, in each of which the number of hydrogen bonds was averaged. The reported SEM was calculated based on these block averages. This analysis allows the internal stabilization of BTD-2 to be compared with its hydration and, for the GdmCl denaturing solution, with direct peptide–GdmCl contacts. The dynamic evolution of the protein–water hydrogen bonds in all 3 solutions is provided in Fig. 10. Overall, although fluctuating over time, the time evolution of the number of protein–water hydrogen bonds reveals a similar pattern across all 3 solvents. This is especially pronounced when focusing on the respective distributions in this graph. All 3 solvents result in similar distributions of protein–water hydrogen bonds, all revealing a main peak followed by a smaller peaks at a slightly lower protein–water hydrogen-bond number. Only the main peak for the protein in NaCl corresponds in average one hydrogen bond less than in KCl and GdmCl, still with no pronounced effect.

**Table 2.** The hydrogen-bond interaction fingerprint of BTD-2 was calculated in the NaCl, GdmCl, and KCl solutions. The SEM from block averaging numbers of internal, protein–water, and direct protein–GdmCl hydrogen bonds are provided. N/A indicates that direct protein–GdmCl interactions are not applicable for the solutions not including GdmCl.

Solvent	Internal	Protein–water	Protein–GdmCl
NaCl	7.8 ± 0.11	46.6 ± 0.49	N/A
GdmCl	8.0 ± 0.10	46.2 ± 0.58	0.5 ± 0.04
KCl	7.5 ± 0.20	47.2 ± 0.60	N/A

BTD-2, baboon theta-defensin-2; SEM, standard error of the mean; GdmCl, guanidinium chloride

**Fig. 11.**
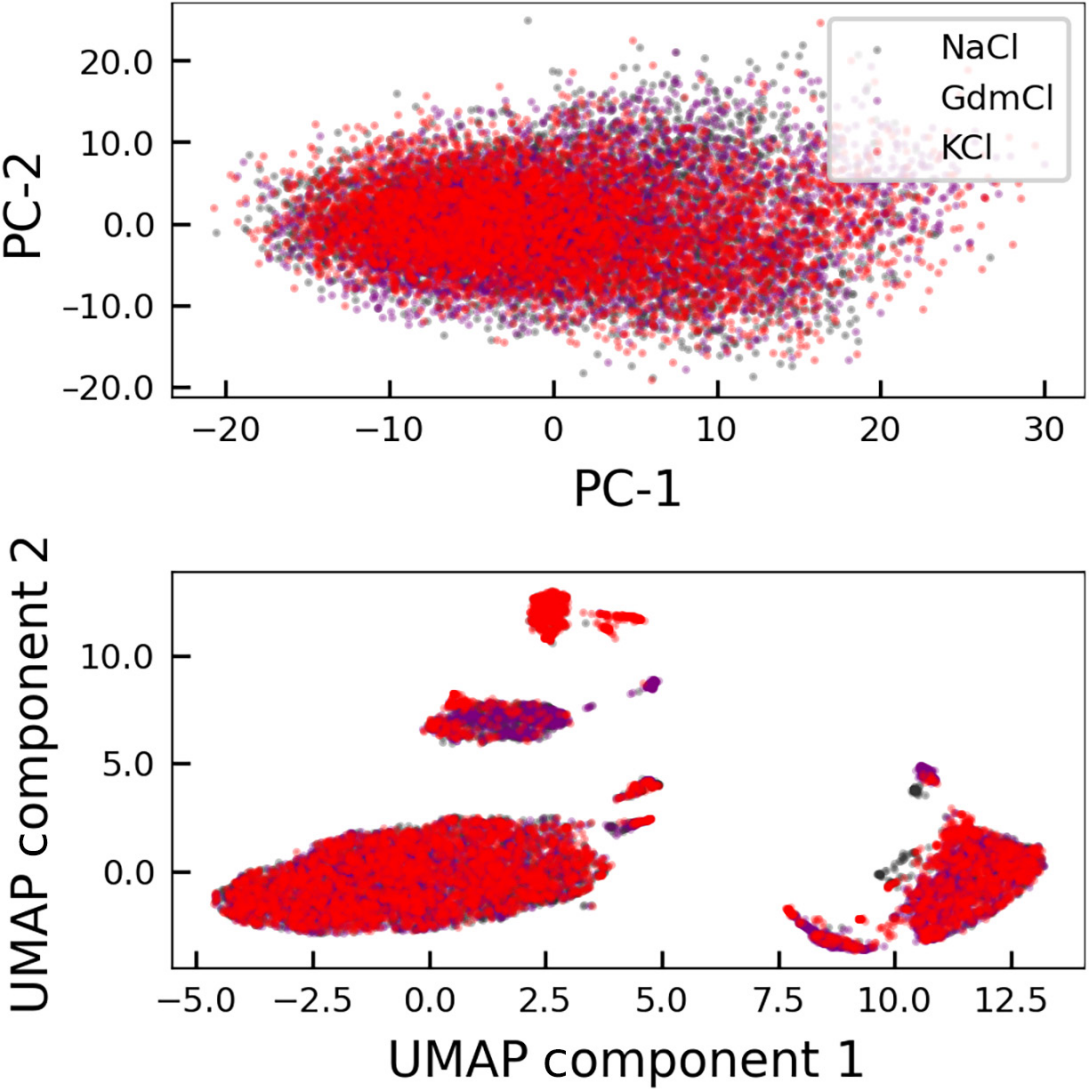
A 2-dimensional (2D) principal component analysis (PCA) projection (top) and a uniform manifold approximation and projection (UMAP) (bottom) representation of the protein trajectory along the 500-ns simulation of wild-type (WT) baboon theta-defensin-2 (BTD-2; ID: 2LYE) in the 3 different solutions, as denoted in the legend.

**Fig. 12.**
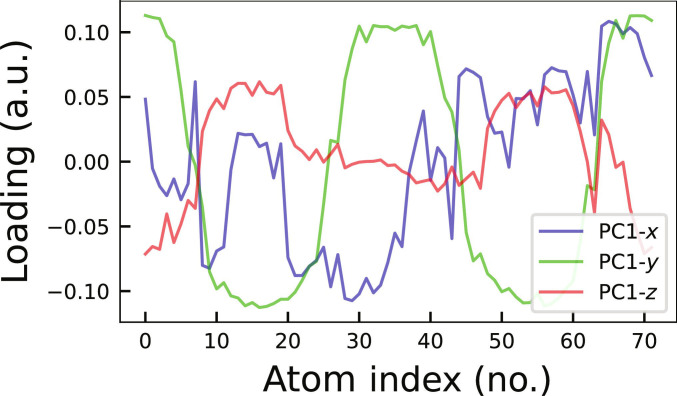
The loadings of the first principal component of the principal component analysis (PCA) shown in Fig. 11. The different atom positions are resolved with respect to their respective cartesian coordinates *x*,*y*,*z*.

All previous results revealed the complex dynamics of BTD-2. In order to overcome this complexity and capture the most important modes of motion while filtering out irrelevant fluctuations, we attempt to reduce the dimensionality of the observed characteristics. To this end, Fig. 11 (top) illustrates the 2-dimensional projection of the trajectory for BTD-2 (ID: 2LYE) in the 3 different solvent environments. The space made of the first 2 PCs in this figure reveals no clusters or any kind of structure differentiating the solvents. The main core of the data lies for all solvents around the same part of the PCA space. This suggests that the interaction of the solutions with the protein does not interfere with the structural conformation of the protein. Note that only the backbone atom positions, and no interaction information, are used for the PCA. Despite the shortcoming of PCA to reveal underlying modes, the loading of the first PC, shown in Fig. 12, unveils a particularly interesting dynamic of the protein. By separating the input feature space by its cartesian coordinate, the loading is a measure of the variance along the respective axis of each atom of the backbone. The variance along the *y*-axis shows that the atoms that can be associated with the turn regions of the protein is larger than that of the sheet regions, in which the disulfide bonds are located. This result is directly correlated and in accordance with the measure of RMSF, shown in Fig. 7. Similar dynamics can be seen in the loadings of the *z*-axis, but far less pronounced. We assign this to the RMSD reducing alignment that was used to preprocess the trajectory data for the PCA. This in combination with the analysis of the sampled conformations over the course of the simulations shows an absence of twisting motions in the protein dynamics and rather a flexing motion of the turn regions. These dynamics have been validated by NMR spectroscopy [8]. As this is a linear dimensionality reduction method, there is a limitation on the kind of dynamics that the reduced space can represent. Therefore, we performed a UMAP on the same data as for PCA to also explore the non-linear dynamics of conformational changes of the protein shown in the top panel of Fig. 11. The non-linear dimensionality reduction in the lower panel confirms the large overlap between the datasets from the different simulations and no solvent-dependent trends. It also resolves, though, different conformational states that the protein assumes over the course of the simulation. Despite the overlap of data, UMAP unveils some rather short-lived states that differentiate the simulations from each other. Inspection of the UMAP representations reveals such short-lived solvent-specific states (focus on the very small areas with only one color in the figure).

### Peptide conformational comparison

We finally tackle the possible differences in the 3 simulated peptides RTD-1, BTD-2, and HTD-2, with IDs 2LYF, 2LYE, and 2LZI in the PDB, respectively. The conformational dynamics and stability of the peptides RTD-1, BTD-2, and HTD-2 were investigated under various ionic conditions, ranging from non-ionic and neutral (0 M) environments to high salt concentrations (0.1, 0.5, 1.0, and 1.5 M KCl). RTD-1 and HTD-2 demonstrated important insensitivity to KCl concentration changes. Regardless of the ionic environment, RTD-1 consistently exhibited a highly fluctuating and dynamic behavior, whereas HTD-2 maintained a remarkably stable and rigid structure across all tested concentrations. In contrast, BTD-2 showed a concentration-dependent stability profile. The peptide displayed enhanced structural stability at 0.5 M KCl and lower concentrations. Investigations for intermediate concentrations (0.75 M) yielded results similar to those observed at 1.0 M, indicating a transition in stability beyond the 0.5 M threshold.

**Fig. 13.**
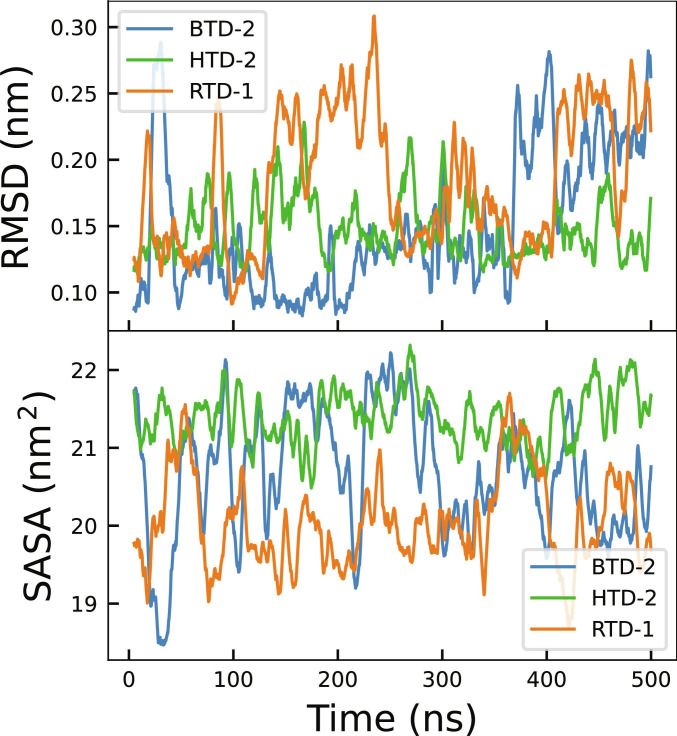
The structural evolution of the baboon theta-defensin-2 (BTD-2), rhesus theta-defensin-1 (RTD-1), and human theta-defensin-2 (HTD-2) peptides in 1 M KCl over the 500-ns-long translocation mapped through the root mean square deviation (RMSD; top) and the solvent-accessible surface area (SASA; bottom).

**Fig. 14.**
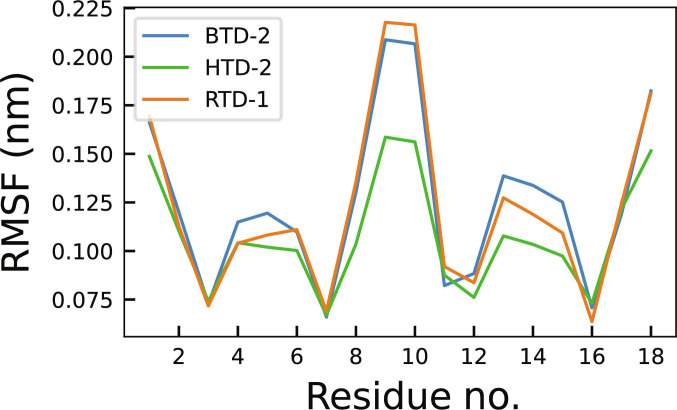
Root mean square fluctuations (RMSFs) per residue for the 3 different peptides in 1 M KCl. For the index, refer to Fig. 2.

In order to highlight the distinct dynamical behavior of these peptides under high ionic stress, a comparative analysis was performed at 1 M KCl in which the trajectories of the peptides clearly differentiate their stability profiles as implied by the RMSD and SASA results in Fig. 13. Consistent with the concentration-independent observations, HTD-2 exhibits the lowest RMSD values, confirming its high rigidity. Conversely, RTD-1 displays larger deviations, indicative of its inherent flexibility. Notably, at this concentration (1 M), BTD-2 also exhibits important structural shifts, contrasting its behavior at lower salt concentrations. The local flexibility of the peptides was further examined through the RMSF in Fig. 14. The respective profiles per residue highlight that HTD-2 maintains low residual fluctuations throughout its backbone, while RTD-1 and BTD-2 show important flexibility in loop regions (residues 8 to 10). For BTD-2, this high-fluctuation pattern in these regions suggest a loss of the rigidity observed at 0.5 M as discussed above.

**Fig. 15.**
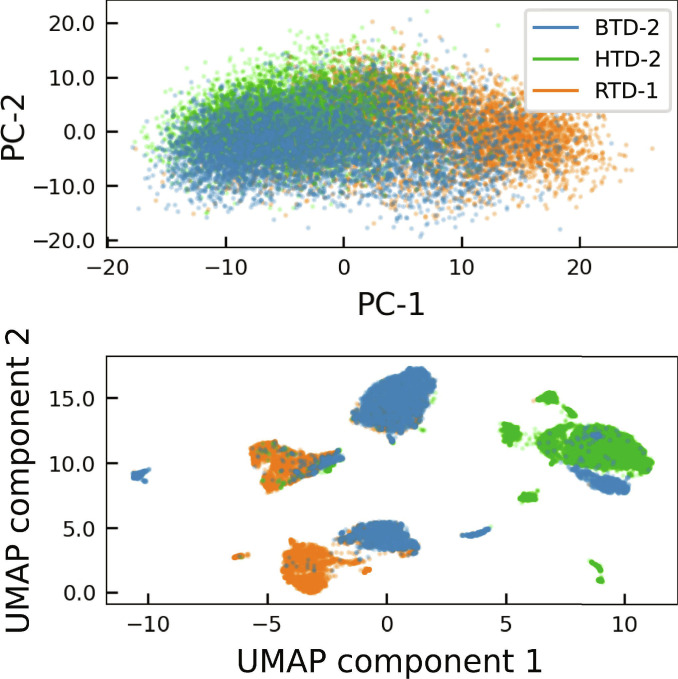
A 2-dimensional (2D) principal component analysis (PCA) projection (top) and a uniform manifold approximation and projection (UMAP) (bottom) representation of the protein trajectories along the 500-ns simulation for all 3 peptides in 1 M KCl colored according to the legend.

Very interesting characteristics were observed through the dimensionality reduction methods as depicted in Fig. 15. In this figure, the 3 peptide trajectories in 1 M KCl are depicted throughout the simulation time. In contrast to the temperature and solvent dependence analysis, here the 3 peptides reveal distinct characteristics in both the PCA and UMAP representations. The PCA space reveals a large overlapping region for the 3 peptides, still part of the space are covered by only one of the peptides denoting distinct trajectories, thus conformation-specific temporal characteristics. These peptide-specific features are emphasized through the UMAP representation. This clearly reveals strong conformational localities, despite the fact that, according to Fig. 2, the amino acid sequences of the 3 peptides are very similar. Accordingly, the 3 peptides might belong to the same family of BTD-2 theta-defensin proteins, providing distinct conformational parts, which can be potentially selectively engineered.

## Conclusions

With the aid of all-atom MD simulations and unsupervised dimensionality reduction techniques, we calculated and analyzed how solvent conditions, temperature, and conformational variability affect theta-defensin proteins. Unlike alpha- and beta-defensins, which can be destabilized by physiological salt concentrations, theta-defensins such as BTD-2 are stabilized by a cyclic cysteine ladder that contributes to their high structural robustness. In our simulations, KCl-containing environments supported the preservation of the BTD-2 scaffold, suggesting that ionic conditions can influence the electrostatic environment around the peptide without disrupting the cyclic fold. GdmCl induced moderate structural perturbations, consistent with its denaturing character, but did not lead to major conformational changes under the conditions studied here. Taken together, these results show that theta-defensin conformations are highly robust, but still sensitive to specific environmental conditions. Note that these results provide a first indication of the solvent’s role in peptide stability, which need to be further investigated in a more focused study of additional solvents and concentrations targeting physiological and industrial environments. The dimensionality reduction schemes were applied here in order to support the unsupervised analysis of MD-generated trajectories. In the case of the theta-defensin proteins studied here, the added value was restrained by their high stability under the selected environmental conditions. Still, such an automatized analysis combined with physics-based simulations is expected to be a powerful toolbox for extracting broad molecular characteristics in diverse conditions and rich conformational variabilities.

We further compared 3 theta-defensin structures and their thermal behavior. The MD simulations indicate that HTD-2 behaves as a robust scaffold under the investigated conditions, whereas RTD-1 shows higher intrinsic flexibility. BTD-2, in contrast, displays a stability profile that is particularly pronounced under KCl-containing conditions. Interestingly, the 3 peptides, although closely related in sequence and stabilized by the same cyclic cysteine ladder motif, display distinct conformational dynamics. This suggests that small sequence differences can influence the accessible flexibility of the theta-defensin scaffold. Importantly, the present simulations do not directly assess antimicrobial activity, membrane binding, target recognition, or functional optimization. Therefore, the results should be further used as structural and dynamical insights on theta-defensin scaffold stability, rather than as direct evidence for improved biological activity. In this context, the preservation of the cyclic cysteine ladder and the sequence- and environment-dependent changes in flexibility may guide future experimental or membrane-based computational studies. Overall, the simulations provide an in-depth molecular picture of theta-defensin stability, solvent sensitivity, and conformational variability. These findings may support future peptide-engineering work by identifying structural features that remain stable across conditions and flexible regions that should be examined further in membrane-relevant systems and functional assays.

## Data Availability

Data generated through the simulations are available under reasonable request.
